# Rice Morphogenesis and Chlorophyll Accumulation Is Regulated by the Protein Encoded by *NRL3* and Its Interaction With NAL9

**DOI:** 10.3389/fpls.2019.00175

**Published:** 2019-02-19

**Authors:** Wei Chen, Zhonghua Sheng, Yicong Cai, Qianlong Li, Xiangjin Wei, Lihong Xie, Guiai Jiao, Gaoneng Shao, Shaoqing Tang, Jianlong Wang, Peisong Hu

**Affiliations:** ^1^State Key Laboratory of Rice Biology, Key Laboratory of Rice Biology and Genetic Breeding of Ministry of Agriculture, China National Rice Research Institute, Hangzhou, China; ^2^The Collaborative Innovation Center of Southern Grain and Oil Crops, Agricultural College of Hunan Agricultural University, Changsha, China

**Keywords:** *nrl3*, narrow and rolled leaf, leaf morphogenesis, chlorophyll accumulation, rice (*Oryza sativa* L.), improvement

## Abstract

Rice yield is closely related to plant leaf shape and chlorophyll content. In this study, we isolated and identified a narrow and rolled leaf mutant, temporarily named *nrl3* with darker green leaves. Histological analysis showed that *nrl3* has a reduced number of vascular bundles and undergoes abnormal abaxial sclerenchymatous cell differentiation. The *NRL3* mutant phenotype was controlled by a single recessive gene, fine-mapped to a 221 kb interval between Indel3 and RM2322 on Chr3. There are 42 ORF in this interval. Sequencing identified an SNP mutant leading to a premature stop in ORF 18, the candidate gene. Bioinformation analysis indicated that *NRL3* encodes a novel protein with unknown function. NRL3 is localized in cytoplasm, membrane and nucleus. Expression analysis of *nrl3* showed that genes involved in chlorophyll synthesis were significantly up-regulated while those involved in chlorophyll degradation and programmed cell death (PCD) were significantly down-regulated. The expression levels of photosynthesis genes were also affected. Y2H and BIFC assays indicated that NRL3 interacts directly with NAL9/VYL to regulate leaf morphology in rice. Thus, *NRL3* plays an important role in leaf morphogenesis and chlorophyll accumulation, and can be used as a new gene resource for constructing improved rice.

## Introduction

Rice is an important crop that provides nutrition and energy to the global population. In recent years, the global demand for rice has increased quickly, but production has risen by less than 1%. Therefore, increasing rice yield is the ultimate goal of rice breeding. Rice leaf is the main photosynthesis organ. Leaf shape affects the efficiency of light capture and energy conversion. Appropriate leaf shape can improve plant photosynthesis efficiency and increase plant yield (Tsukaya, 2006; Micol, 2009).

Many studies have characterized genes which control the rolling and width of rice leaves. The process of leaf development in rice has been divided into four parts: leaf primordia formation, polarity establishment, tissue differentiation and leaf extension (Fan and Liang, 2014). Establishment of polarity is an important process that affects leaf morphology by regulating the adaxial (inward) and abaxial (outward) rolling of leaves (Fan and Liang, 2014). Research in *Arabidopsis thaliana* and maize showed that transcription factors and small RNAs are involved in regulation during establishment and maintenance of leaf adaxial–abaxial axis polarities (Moon and Hake, 2011). HD-ZIPIII family genes, such as *PHB*, *PHV* (McConnell et al., 2001), and *REV* (Otsuga et al., 2001), negatively regulated by microRNA miR165 and miR166, have been proved to regulate organ polarity, vascular development, and meristem function (Mallory et al., 2004; Kidner and Martienssen, 2004; Prigge et al., 2005). Further studies of *PHABULOSA* (*PHB*), *PHAVOLUTA* (*PHV*), and *REVOLUTA* (*REV*) in *A. thaliana* have shown that these genes exhibit a polar distribution expression pattern in the lateral organs which as an important regulatory factor for the establishment and maintenance of the adaxial features of these organs (Prigge et al., 2005). In addition, the *YAB2* and *YAB3* genes (Eshed et al., 2004) belonging to the YABBY family in rice, and *MILKWEED POD1* (*MWP1*) (Candela et al., 2008) belonging to the KANADI family in maize, play important roles in the development of abaxial cells.

There is substantial evidence that defects in the establishment of polarity have a major impact on leaf rolling. Recently, a number of genes involved in the establishment of leaf polarity in rice have been identified. For example, *SHALLOT-LIKE1* (*SLL1*), part of the KANADI family and having homology with KANADI family genes of *A. thaliana*, encodes a SHAQKYF class MYB family transcription factor regulating programmed cell death (PCD) of mesophyll cells, and affecting abaxial features of leaves (Zhang et al., 2009). *OsAGO7* encodes an Argonaute (AGO) family protein, over-expression of which in rice causes upward rolling leaves (Shi et al., 2007). *ADL1* gives rise to a plant-specific calpain-like cysteine proteinase which participates in leaf adaxial–abaxial axis maintenance, which the mutant showed abaxially rolled leaves (Hibara et al., 2009).

In addition to the establishment of polarity, the shape of bulliform cells also has an important role in rolling of leaves (Alvarez et al., 2008). *Rolling-leaf 14* (*RL14*) encodes a 2OG-Fe (II) oxygenase family protein which regulates curling of leaves by affecting the formation of secondary walls. Mutants with an altered secondary wall composition exhibit changes in the shape of the bulliform cells, which affects water transport in the leaf (Fang et al., 2012). *SRL1* encodes a Putative Glycosylphosphatidylinositol-Anchored Protein that inhibits the formation of bulliform cells by negatively regulating the expression of genes encoding vacuolar H^+^-ATPase subunit and H^+^-pyrophosphatase, thereby modifying leaf curl (Xiang et al., 2012).

Cell division and expansion are essential phases in the conversion of leaf primordia to mature leaves (Gonzalez et al., 2012), determining leaf size (Fujino et al., 2008). Many mutants showing abnormal leaf size have been characterized, caused by defects in cell division and cell expansion. For instance, *Slender Leaf 1* (*SLE1*) encodes cellulose synthase-like D4, regulating cell proliferation during M phase and participated in cell wall formation, the mutant of which showed narrow and rolling leaves (Li M. et al., 2009; Wu et al., 2010; Yoshikawa et al., 2013). *NAL2* and *NAL3* are paralogs which encode the same *OsWOX3A* transcriptional activator. Their double mutant *nal2/3* showed narrow and rolling leaves, and fewer veins (Cho et al., 2013, 2016). *NAL1* encodes a trypsin-like serine/cysteine protease, affecting polar auxin transport and vascular patterns of rice, and participates in cell division (Qi et al., 2008; Jiang et al., 2015).

In the present study, we isolated and identified a narrow and rolled leaf mutant, *nrl3*. Compared with the wild-type, *nrl3* showed darker green leaves at the tillering stage and with shorter panicles and longer, narrower seeds. Histological analysis showed that *nrl3* had a decreased number of vascular bundles and defects of the abaxial sclerenchymatous cells which, respectively, caused leaf narrowed and rolling in rice. Map-based cloning showed that *NRL3* encodes a novel protein with unknown function that can interact with *NAL9/VYL* directly to regulate leaf morphology in rice. In depth analysis of NRL3 can enhance our understanding of leaf morphogenesis and provides new genetic resources for improving rice.

## Materials and Methods

### Plant Materials and Growth Conditions

The *nrl3* line was from a library of *Oryza sativa* subsp. *indica* cv. Zhongjiazao 17 (YK17) mutants obtained by EMS treatment. The F_2_ population for gene mapping was generated by crossing between *nrl3* and tropical *japonica* rice variety D50. Plants were grown under natural conditions in the experimental fields of China National Rice Research Institute in Fuyang, Hangzhou, China.

### Measurement of LRI and Net Photosynthetic Rate

Leaf rolling index (LRI) was determined for the upper leaf by measuring L_w_ (the maximum width of the expanded leaf blade) and L_n_ (the natural distance of the leaf blade margins at the same position). Then LRI(%) = (L_w_-L_n_)/L_w_^∗^100 (Shi et al., 2007). Photosynthetic rate and transpiration rate were measured at the heading stage from 12: 00 to 13: 00 with a Li-6400 portable photosynthesis apparatus. Measurements of LRI, net photosynthetic rate and transpiration rates of wild-type and the *nrl3* mutant were repeated at least three times, each replication used five independent plants.

### Map-Based Cloning of the *NRL3* Gene

To map the *NRL3* locus, plants with narrow and rolling leaves were selected from the F_2_ population. First, we used both parents and 20 F_2_ individuals with the mutant phenotype for linkage analysis of *NRL3*. More than 116 polymorphic SSR markers evenly distributed on the whole rice genome were employed. Then a further 936 recessive individuals with the mutant phenotype were selected from the F_2_ population to fine map the *NRL3* locus. SSR and Indel markers were developed based on nucleotide polymorphisms between YK17 and D50 in the corresponding regions (Supplementary Table S1). PCR products of candidate genes were amplified from both *nrl3* and YK17 genomic DNA for sequencing. The sequencing results were analyzed using DNAMAN.

### Scanning Electron Microscopy

The leaves of the wild-type and the *nrl3* mutant at the heading stage were collected, fixed in 2.5% glutaraldehyde solution at 4°C overnight, and rinsed with 0.1 M phosphate buffer (pH 7.4). Then, the samples were dehydrated in a graded ethanol series, 20 min for each step, followed by substitution with isoamyl acetate. The samples were critical-point dried and sputter-coated with gold. The samples were observed and photographed using a scanning electron microscopy (XL30, Philips, United States).

### Paraffin Sections

The fresh tissues were first fixed in formalin-glacial acetic acid-alcohol solution (FAA: 70% ethanol, acetic acid, formaldehyde; 16:1:1) for 24–48 h. The fixed tissues were dehydrated by passage through different concentrations of alcohol (75%, 85%, 95%, and absolute ethanol) and finally embedded in paraffin. Cross-sections (5–12 μm) were cut and stained with safranin and then observed under a microscope (3D HISTECH).

### RNA Extraction and qRT-PCR Analysis

Total RNA was extracted from different plant tissues (root, stem, leaf, leaf sheath, panicle, and seeds) of wild-type and *nrl3* using Trizol reagent (Life Technologies). The first strand cDNA was reverse-transcribed by using First Stand cDNA Synthesis Kit (TOYOBO). PCR program was: 95°C for 30 s, 95°C for 5 s, 58°C for 30 s, 72°C for 15 s, totally 40 cycles. Quantitative real-time PCR determination was based on the SYBR Green Real-time PCR Master Mix (Toyobo). Primers for quantitative real-time PCR (qRT-PCR) analysis are listed in Supplementary Table S1. The rice actin gene (*Os03g0718150*) was used as internal control. All the materials used for RNA extraction were from mixing three independent plants.

### Plasmid Construction and Rice Transformation

For over-expression of the *nrl3* mutant, the wild-type *NRL3* cDNA sequence from YK17 was cloned using infusion primers: 5′-TTACTTCTGCACTAGGTACCATGGGTTTCATGTCAGCGA-3′ and 5′-TGGCTAGCGTTAACACTAGTCTGGGCACGATATGCAGCC-3′, and the PCR product was constructed into binary vector pCAMBIA1390 driven by the *UBIQUTIN1* promoter (Clontech^1^). The CRISPR target of NRL3 (CAGCTCTGCGCCCAAGCTCGCGG) was cloned into Cas9/gRNA Vector (VK005-01, Beijing Viewsolid Biotech Co., Ltd.^2^). These plasmids were introduced into *Agrobacterium tumefaciens* strain EHA105. The overexpression vector was transformed into the *nrl3* mutant, and the CRISPR/Cas9 vector was transformed into wild-type plants. Genotypes of the independent transgenic lines were determined by PCR amplification of the specific transgenic fragment (all primers used for plasmid construction are listed in Supplementary Table S1).

### Subcellular Localization of *NRL3*

The full-length coding sequence of wild-type NRL3 from YK17, without a termination codon, was amplified by PCR and subcloned into pAN580 vector to obtain the NRL3-GFP construct. The fusion plasmid was transiently co-expressed in rice protoplasts using polyethylene glycol according to the protocols described previously (Chen et al., 2006). After incubating for 36 h at 28°C in the dark, GFP fluorescence in the protoplast cells was detected using a Zeiss LSM510 laser scanning confocal microscopy (Karl Zeiss, Jena, Carl Zeiss AG, Germany). The free pAN580 vector was used as control.

### Determination of Chlorophyll Content

Fresh leaves of wild-type and *nrl3* rice plants at heading stage were used to determine chlorophyll content according to the previous described method (Wu et al., 2007). Aliquots of 0.2 g fresh leaves from wild-type and *nrl3* were cut into 1–2 mm pieces, immersed in 25 ml ethanol, placed in the dark for 48 h, and then centrifuged at 8000 *g* for 10 min. A DU800 spectrophotometer (Beckman Coulter, United States) was used to measure absorption of the supernatant at 665 and 649 nm.

### Yeast Two-Hybrid Analysis

The full-length coding region of *NRL3* without a termination codon was amplified by PCR and cloned into pGBKT7 vector. Two-Hybrid Library Screening Using Yeast Mating was used to find protein interactions with NRL3, applying the protocol described by the manufacturer with little adjustments (Matchmaker^®^ Gold Yeast Two-Hybrid System User Manual, PT4084-1 (092413), Clontech). After sequencing for identification of the interacting protein, full-length cDNA of VYL without a termination codon (also named NAL9, *LOC_Os03g29810*) was amplified using infusion primers for construction into PGADT7 vector. Yeast transformation and selection procedures were carried out according to the manufacturer’s instructions [Yeastmaker^TM^ Yeast Transformation system 2 User Manual, PT1172-1 (PR8Y2629), Clontech].

### BIFC Assay

The full-length cDNA fragments of NRL3 and VYL without stop codons were, respectively, cloned into binary vectors pSPYNE and pSPYCE to obtain pNRL3-YFPNE and pVYL-YFPCE. These plasmids were transiently expressed in tobacco leaves using *A. tumefaciens* strain EHA105 following the method described by Xiang et al. (2012). A confocal laser scanning microscope (Zeiss LSM710) was used to detect YFP fluorescent signals after 84 h post-transfection. Primers used in this assay are listed in Supplementary Table S1.

## Results

### Characterization of *nrl3* Mutants

**FIGURE 1:**
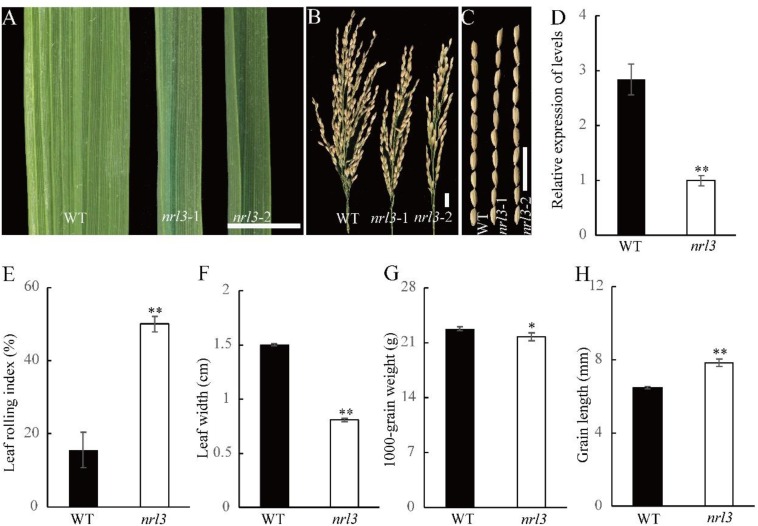
Phenotype the *nrl3* mutant. **(A)** Upper leaf of WT, *nrl3*-1, and *nrl3*-2. **(B)** Panicle length of WT, *nrl3*-1, and the *nrl3*-2 mutant. **(C)** Grain length of WT, *nrl3*-1, and *nrl3*-2. Bar: 1 cm **(A–C)**. **(D)** Expression analysis of *NRL3* in WT and *nrl3* mutant plants. RNA was isolated from leaves at tillering stage of WT and *nrl3* of three independent plants. Data are shown as means ± SD for three biological replicates. **(E)** Leaf rolling index (LRI) of the flag leaf of WT and *nrl3* of three independent plants. **(F)** Leaf width of the flag leaf of WT and the *nrl3* mutant plant of three independent plants. Data are shown as means ± SD for three biological replicates. **(G)** Thousand-grain weight of WT and *nrl3*. **(H)** Grain length of WT and *nrl3*. Data are shown as mean ± SD from three biological replicates, each replication was not less than 200 seeds. Asterisks indicate statistical significance as determined by Student’s *t*-test (^∗^*P* < 0.05, ^∗∗^*P* < 0.01).

The mutant *nrl3* was identified from a library produced using ethyl methanesulfonate (EMS) on an *indica* rice variety Zhongjiazao 17 (YK17). The *nrl3* plants exhibited narrow and rolled leaves over the whole duration of growth. They had darker green leaves at the tillering stage which became more obvious with plant growth (Figure 1A and Supplementary Figures S5A,B). Statistical analysis showed that the LRI of *nrl3* (50%) was over three times that of wild-type (15%) (Figure 1E), and the flag leaf width of *nrl3* was reduced by approximately 47% (Figure 1F). Investigation of agronomic traits revealed no obvious differences in the plant height and effective tillers number between wild-type and *nrl3* (Supplementary Figures S1A,C), but panicle length was significantly decreased in *nrl3* (Figure 1B and Supplementary Figure S1B). Finally, the grain width and 1000-grain weight of *nrl3* were significantly decreased (Figure 1G and Supplementary Figure S1D), while the grain length of *nrl3* was significantly increased (Figure 1C,H) compared with the wild-type. Therefore, mutation in *NRL3* leads to multiple changes in plant agronomic traits.

### Defects in Sclerenchymatous Cells and Reduction in the Number of Vascular Bundles Cause the Rolled and Narrow Leaf in *nrl3*

**FIGURE 2:**
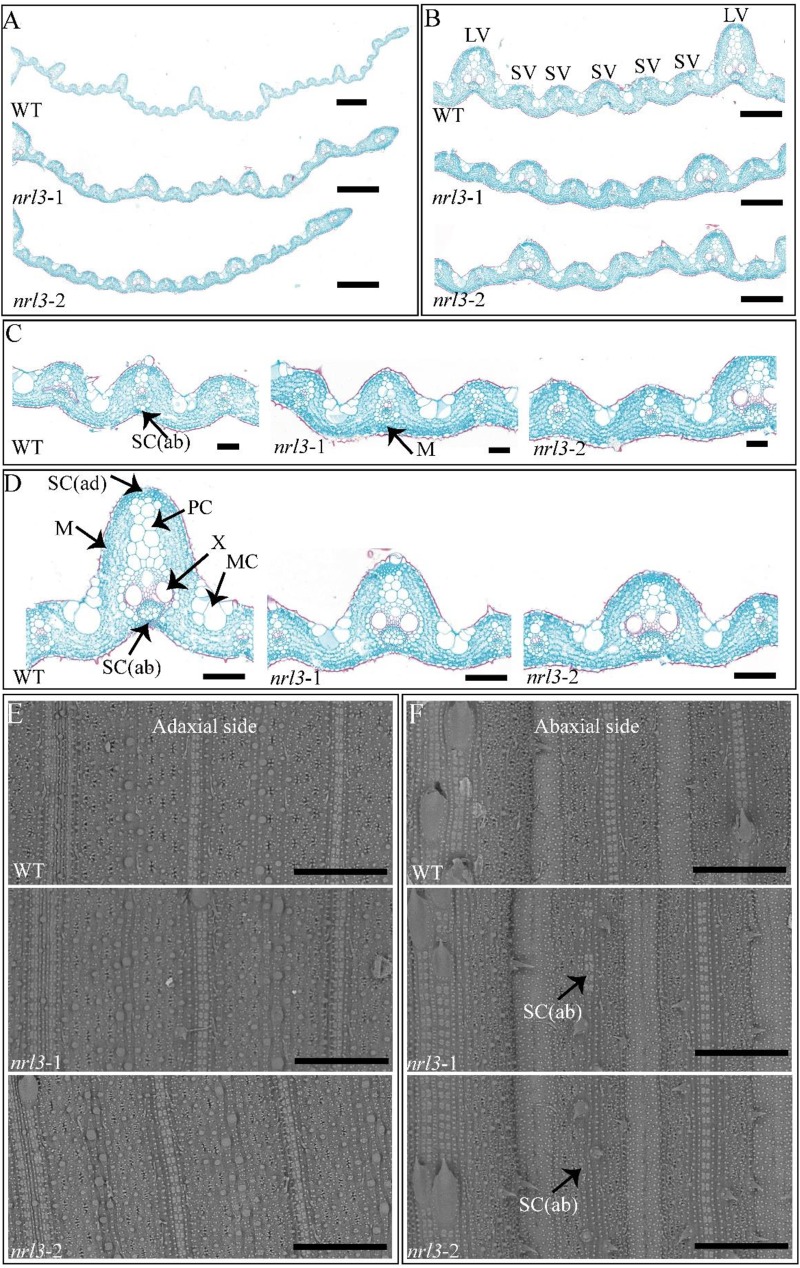
Cytological analysis of WT and nrl3. **(A–D)** Cross sections of leaves at the heading stage from WT, *nrl3*-1, and *nrl3*-2. **(E,F)** Scanning electron microscopy of the adaxial and abaxial sides of WT, *nrl3*-1, and *nrl3*-2 leaves. LV, large veins; SV, small veins; SC(ab), abaxial sclerenchyma cell; SC(ad), adaxial sclerenchyma cell; MC, motor cell; M, mesophyll cell; X, xylem; PC, parenchymal cell; Bar: 500 μm **(A)**, 200 μm **(B)**, 50 μm **(C)**, 100 μm **(D)**, 300 μm **(E,F)**.

To explore the reasons for the narrow and rolled leaves in *nrl3* mutant, we carried out an analysis of cross-sections in the flag leaf. Compared with the wild-type, the *nrl3* had fewer large and small veins. There were on average only six large veins in the *nrl3* mutant, half the number in the wild-type (Figure 2A). Furthermore, in *nrl3* mutant there were on average three small veins between each two neighboring large veins, compared to six in the wild-type (Figure 2B). Observed the cross-sections, we found that the development of abaxial sclerenchymatous cells was defective in the region of some small veins in *nrl3* mutant, although the adaxial sides were normal (Figure 2C). In addition, we found that the air cavity and parenchymal cell of the *nrl3* leaves differed from the wild-type. The mutant displayed the number of parenchyma cells on the large veins was decreased (Figure 2D). To confirm our results, we observed the abaxial side and adaxial side of the leaves by scanning electron microscopy (SEM). This showed defects in the sclerenchymatous cells on the abaxial side of the *nrl3* leaves, while the adaxial side was similar to the wild-type (Figure 2E,F).

### Map-Based Cloning of *NRL3*

**FIGURE 3:**
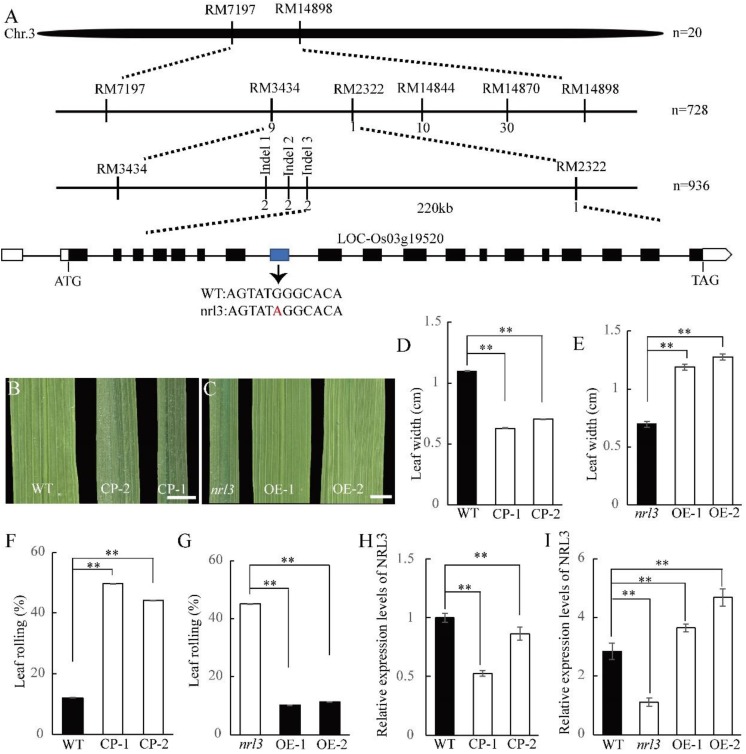
Map-based cloning of *NRL3*. **(A)** Map of *NRL3* locus in a 221 kb region between markers Indel 3 and RM2322 on chromosome 3 (Chr3). **(B)** The leaf phenotype of knock-out transgenic plants (CP). Bar: 1 cm. **(C)** The phenotype of overexpression transgenic plants (OE). Bar: 1 cm. **(D,E)** Upper leaf width of knock-out transgenic plants **(D)** and overexpression transgenic plants **(E)**. **(F,G)** Upper leaf rolling in knock-out transgenic plants **(F)** and overexpression transgenic plants **(G)**. **(H)** Expression levels of *NRL3* in knock-out transgenic plants. **(I)** Expression level of *NRL3* in overexpression transgenic plants. RNA was isolated from leaves at tillering stage of wild-type, *nrl3* and transgenic plants. Data are shown as means ± SD from three replicates. The asterisks indicate statistical significance between the WT and the *nrl3* mutant, as determined by Student’s *t*-test (^∗∗^*P* < 0.01).

In order to identify the inheritance of *NRL3*, map-based cloning was conducted using a F_2_ population produced by crossing the *nrl3* with D50 (tropical *japonica* variety). Genetic analysis showed that the plant number ratio for wild-type and *nrl3* individuals in the F_2_ population was 3:1 (185 wild-type plants and 68 mutant plants; *x*^2^ = 0.4756 < *x*^2^_0.05,1_ = 3.84), indicated that the phenotype of *nrl3* is controlled by a single recessive gene. The locus of *NRL3* was first mapped on the short arm of chromosome 3 between the simple sequence repeat (SSR) markers RM7197 and RM14898 (Figure 3A). To fine-map *NRL3*, 936 F_2_ individuals with the *nrl3* phenotype were chosen for genotyping, which further narrowed the mapping interval down to a 221-kb region between Indel marker Indel 3 and SSR marker RM2322 (Figure 3A). According to the Rice Genome Annotation Project database^3^, there are 42 ORFs in this interval, which were selected for sequencing analysis. We found a single substitution of a guanine (G) with an adenine (A) on the 8th exon of *LOC_Os03g19520* (Figure 3A), but no mutation in any of the other predicted ORFs. This G→A substitution leads to the premature termination of NRL3 (Supplementary Figure S2).

To confirm *LOC_Os03g19520* is the target gene of *NRL3*, vectors carrying the full-length coding region of *NRL3* driven by the *UBIQUTIN1* promoter, or knock-out vector using the Crispr/Cas9 system, were constructed and introduced into *nrl3* and wild-type, respectively. We found all of the over-expression plants were able to rescue the narrow and rolled leaf phenotype of *nrl3*, with increased leaf width, decreased LRI, and a lighter green color (Figure 3C,E,G,I). Furthermore, after knock-out of *LOC_Os03g19520* in wild-type, the resulting phenotype is similar to *nrl3* (Figure 1D, 3B,D,F,H). Thus *LOC_Os03g19520* was confirmed as the target gene of *NRL3*. Protein sequence analysis showed *NRL3* encoded a novel protein with unknown function. Phylogenetic analysis revealed that NRL3 protein may have a conserved function in both monocotyledons and dicotyledons (Supplementary Figure S4).

### Expression Pattern and Subcellular Localization of NRL3

**FIGURE 4:**
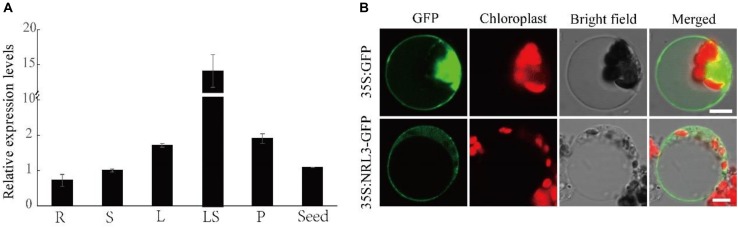
Expression pattern and subcellular localization of NRL3. **(A)** qRT-PCR analysis of *NRL3* expression level in various tissues. R, root; S, stem; L, leaf; LS, leaf sheath; P, panicle. **(B)** Subcellular localization of *NRL3* in rice protoplast cells. Free 35S: GFP, which localized to the cytoplasm, membrane and nucleus, was used as a control; Full-length NRL3 fusion protein (35 S: NRL3-GFP) similarly localized to the cytoplasm, membrane and nucleus. At 36 h after transformation, rice protoplast cells were observed using a confocal laser scanning microscope. GFP signals (Green), chlorophyll autofluorescence (Red), bright-field images, and the merged images of GFP signal and chlorophyll signals are shown in each panel. Bars: 5 μm.

Quantitative real-time PCR analysis suggested that *NRL3* was constitutively expressed in all tested organs, with the highest levels in leaf sheaths (Figure 4A). NRL3-GFP fusion protein was ubiquitous in protoplasts, suggesting that NRL3 may be found in the nucleus, membrane and cytoplasm. The green fluorescence of NRL3-GFP was coincident with the autofluorescence of chlorophyll (Figure 4B).

### *NRL3* Negatively Regulates Chlorophyll Synthesis and Affects Chlorophyll Degradation

**FIGURE 5:**
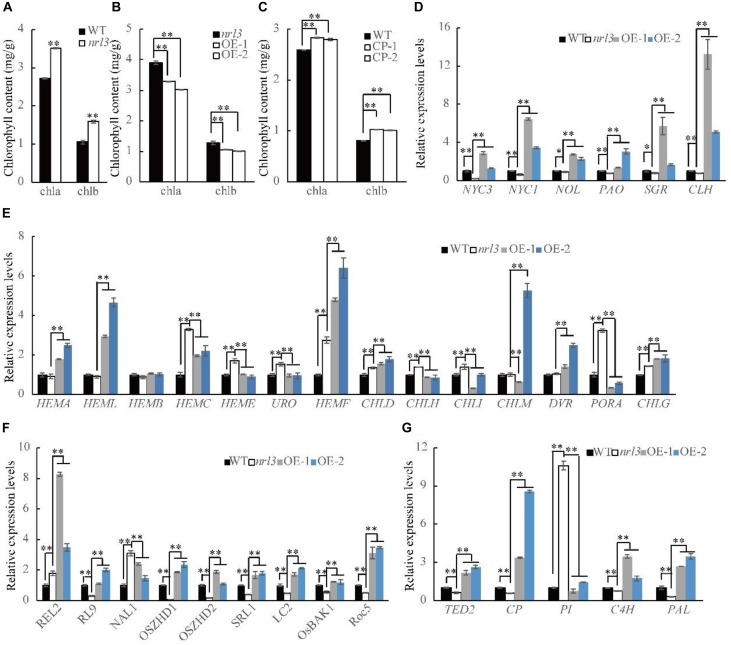
Chlorophyll contents and the transcriptional changes of genes in WT, *nrl3* and transgenic plants. **(A)** Chlorophyll content of leaves in WT and *nrl3* mutant at heading stage. Chla, Chlorophyll a; Chlb, Chlorophyll b. **(B)** Chlorophyll content of leaves in *nrl3* and OE mutant at heading stage. **(C)** Chlorophyll content of leaves in *nrl3* and CP mutant at tillering stage. **(D)** Relative expression of genes involved in chlorophyll degradation in WT, *nrl3*, OE-1, and OE-2. **(E)** Relative expression of genes involved in chlorophyll biosynthetic genes in WT, *nrl3*, OE-1, and OE-2. **(F)** Expression levels of genes associated with leaf morphology development in WT, *nrl3*, OE-1, and OE-2. **(G)** Relative expression levels of PCD genes. RNA was isolated from leaves at heading stage of WT, the *nrl3* mutant, OE-1, and OE-2. Data are shown as means ± SD for three biological replicates. The asterisk indicates statistical significance determined by Student’s *t*-test (^∗^*P* < 0.05; ^∗∗^*P* < 0.01).

Leaves of *nrl3* were darker green, and the contents of chlorophyll a (chla) and chlorophyll b (chlb) were significantly increased in its flag leaves (Figure 1B, 5A). Furthermore, chlorophyll content was decreased in the *NRL3* over-expression lines, but increased in the knock-out lines (Figure 5B,C). In addition, *nrl3* exhibited a significantly lower photosynthetic rate and higher transpiration rate than the wild-type, and the expression levels of photosynthesis-related genes was significantly down-regulated (Supplementary Figures S3A–C). Therefore, the expression of chlorophyll biosynthesis-related genes was analyzed *via* qRT-PCR. Compared with wild-type the expression in *nrl3* of most chlorophyll biosynthetic genes was significantly up-regulated, including: *HEMC* (Porphobilinogen deaminase, *LOC_Os02g07230*), *HEME* (Uroporphyrinogen decarboxylase 1, *LOC_Os01g43390*), *URO* (Uroporphyrinogen decarboxylase 2, *LOC_Os03g21900*), *HEMF* (Coproporphyrinogen III oxidase, *LOC_Os04g52130*), *CHLD* (Mg chelatase D subunit, *LOC_Os03g59640*), *CHLH* (Mg chelatase H subunit, *LOC_Os03g20700*), *CHLI* (Mg chelatase I subunit, *LOC_Os03g36540*), *PORA* (Protochlorophyllide reductase A, *LOC_Os04g58200*), and *CHLG* (Chlorophyll synthase, *LOC_Os05g28200*). The other genes we examined were unchanged in *nrl3*: *HEMA* (Glutamyl-t RNA reductase, *LOC_Os10g35840*), *HEML* (Glutamate-1-semialdehyde 2,1-aminomutase, *LOC_Os08g41990*), HEMB (Delta-aminolevulinic acid dehydratase, *LOC_Os06g49110*), CHLM (Mg-protoporphyrin IX methyltransferase, *LOC_Os06g04150*), and DVR (Divinyl chlorophyllide a 8-vinyl-reductase, *LOC_Os03g22780*) (Figure 5E). In addition we examined the expression of chlorophyll degradation-related genes, and found the following groups significant decreased in *nrl3* and significant up-regulated in OE-1 and OE-2 (Figure 5D): *NYC1* (Chlorophyll b reductase gene, *LOC_Os01g12710*), *NYC3* (α/β hydrolase-fold family protein, *LOC_Os06g24730*), *NOL* (Chlorophyll b reductase gene, *LOC_Os03g45194*), *PAO* (pheophorbide a oxygenase, *LOC_Os03g05310*), SGR (stay green gene, *LOC_Os09g36200*), and CLH (chlorophyllase-2, *LOC_Os10g28370*).

### Genes Associated With Leaf Morphology Development Were Affected in *nrl3*

Quantitative real-time PCR analyses were performed to investigate the relationship between *NRL3* and other leaf morphology regulatory genes. The results showed that *REL2* (rolled and erect leaf 2, Yang et al., 2016) and *NAL1* (trypsin-like serine/cysteine protease, Qi et al., 2008; Jiang et al., 2015) was significantly up-regulated in the *nrl3*. In contrast, *RL9* (a GARP protein gene, Yan et al., 2008), *OsZHD1* and *OsZHD2* (Zn-finger transcription factor, Figueiredo et al., 2012; Xu et al., 2014), *SRL1* (glycosylphosphatidylinositol-anchored protein, Xiang et al., 2012), *LC2* (vernalization-insensitive-3-like protein, Wang et al., 2013), *ROC5* (homeodomain leucine zipper class IV gene, Zou et al., 2011), and OsBAK1 (SERK-family receptor-like protein kinase, Li D. et al., 2009) were significantly down-regulated in *nrl3* (Figure 5F). In addition, compared to *nrl3*, the expression of most genes in OE-1 and OE-2 was up-regulated (Figure 5F).

### *nrl3* Causes Leaf Rolling by Affecting PCD of Abaxial Mesophyll Cells

Previous studies have shown that PCD is involved in the formation of sclerenchymatous cells. Thus, we examined in wild-type and *nrl3* the expression levels of rice genes which share highly identity with TE-PCD marker genes from *Zinnia* or other species. Results showed that *TED2* (*LOC_Os10g41170*), *CP* (*LOC_Os08g44270*), *C4H* (*LOC_Os05g25640*), and *PAL* (*LOC_Os05g35290*) were significantly down-regulated, while *PI* (*LOC_Os05g06780*) was significantly up-regulated (Figure 5G). In addition, compared to *nrl3* the expression of most genes in OE-1 and OE-2 was significant up-regulated (Figure 5G). According to previous studies we know that *TED2* is the marker gene of stage two of PCD, with *CP*, *C4H*, *PI*, and *PAL* marking stage three. Together, these results indicate that the effect of *NRL3* on development of sclerenchymatous cells may be via regulation of PCD in abaxial mesophyll cells.

### NRL3 and VYL Proteins Form a Complex

**FIGURE 6:**
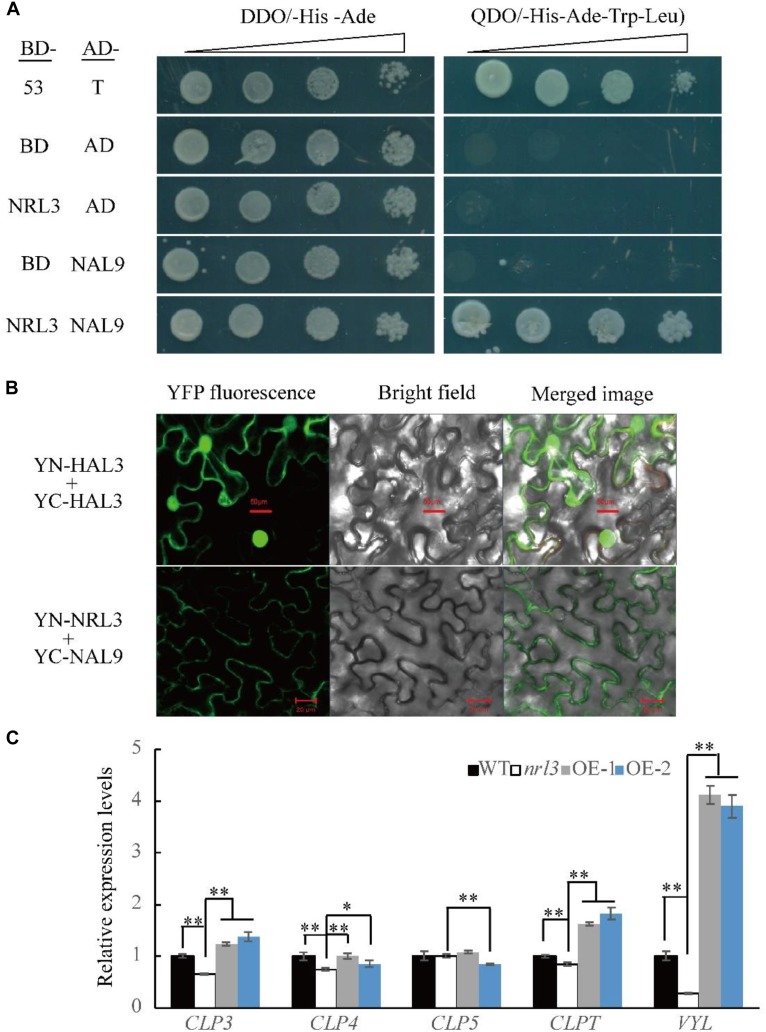
NRL3 interaction with NAL9/VYL. **(A)** Yeast two-hybrid assays showed interaction between NRL3 and VYL. The interactions between PGBKT7-53 and PGADT7-T, PGBKT7, and PGADT7 were used as the positive control and negative control, respectively. **(B)** BIFC assay confirmed the interaction of NRL3 and VYL (Bar = 20 μm). The interaction of YN-HAL3 and YC-HAL3 was used as positive control (Bar = 50 μm). **(C)** Expression of *CLP3*, *CLP4*, *CLP5*, *CLPT*, and *VYL*/*NAL9* in WT, *nrl3*, OE-1, and OE-2. Data are means ± SD for three biological replicates. The asterisk indicates the difference determined by Student’s *t*-test (^∗^*P* < 0.05; ^∗∗^*P* < 0.01).

**FIGURE 7:**
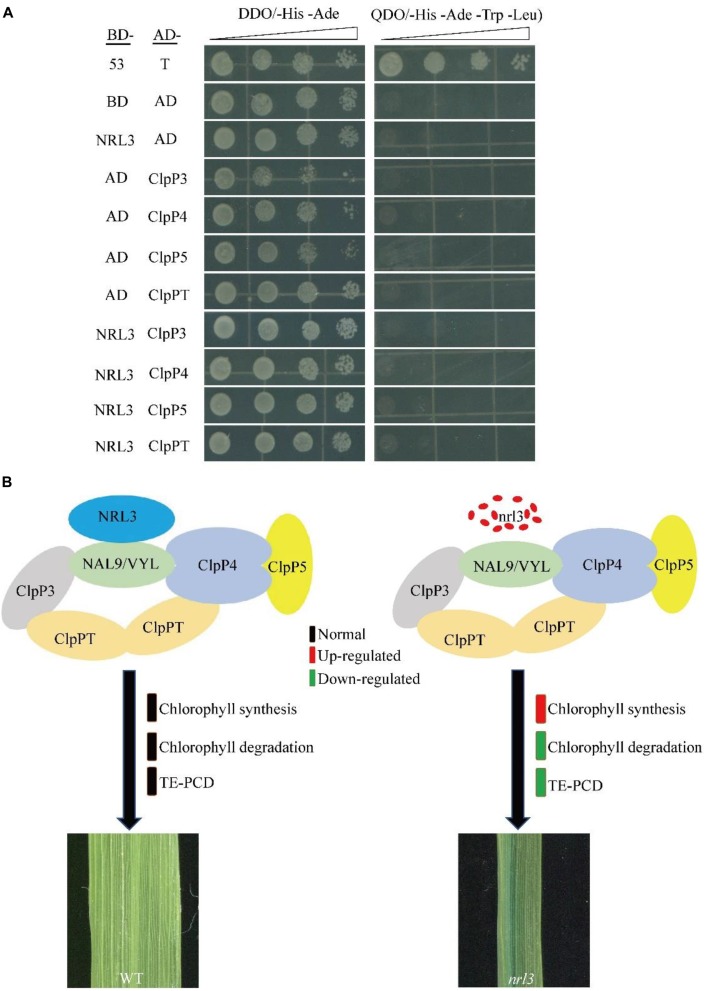
Y2H analysis of NRL3, ClpP3, ClpP4, ClpP5, and ClpPT, and our model showing the role NRL3 plays in regulating leaf development and chlorophyll degradation. **(A)** Yeast two-hybrid assays showing the absence of interaction between NRL3 and ClpP3, ClpP4, ClpP5, and ClpPT. The interactions between PGBKT7-53 and PGADT7-T, PGBKT7, and PGADT7 were used as the positive control and negative control, respectively. **(B)** Model showing the important role NRL3 plays in regulating leaf development and chlorophyll degradation.

*NRL3* gene was predicted to encode a protein of unknown function. To explore its mode of action we screened the yeast two-hybrid (Y2H) library of rice seedlings. Using *NRL3* as bait, we screened and sequenced many independent fragments interacting with NRL3. The sequencing results showed that the NAL9/VYL gene matched with partial clones. In rice, *VYL* encodes ATP-dependent *Clp* protease proteolytic subunit protein, which plays an important role in rice chloroplast biosynthesis and leaf development. The *vyl* mutant displayed narrow foliage, pale green leaf phenotypes throughout the growth period (Dong et al., 2013; Li et al., 2013). The yeast two-hybrid assay (Y2H) showed that NRL3 directly interacted with VYL (Figure 6A). In addition, bimolecular fluorescence complementation (BIFC) showed that NRL3 and VYL interact on the membrane (Figure 6B). In summary, there is a strong interaction between NRL3 and VYL. VYL forms a complex with ClpP4, ClpP5, ClpP, and ClpP3, and plays important roles in the biosynthesis of chloroplasts and in leaf development, while *ClpP4*, *ClpP5*, *ClpT*, *ClpP3*, and *VYL* have similar expression patterns (Dong et al., 2013). Consequently we examined the expression levels of *ClpP4*, *ClpP5*, *ClpT*, *ClpP3*, and *VYL* in WT, *nrl3*, OE-1, and OE-2, and we found that expression of *ClpP4*, *ClpT*, *ClpP3*, and *VYL* was significantly down-regulated in *nrl3* and significantly up-regulated in OE-1 and OE-2 (Figure 6C). In addition, the Y2H results showed that NRL3 did not interact with ClpP3, ClpP4, ClpP5, or ClpPT (Figure 7A).

## Discussion

Leaf morphology is an important agronomic trait. Changes in leaf shape impact on photosynthesis efficiency, with consequences for rice yield. Mutations of leaf morphology have been divided into two main categories, narrow leaves and rolled leaves. Factors affecting leaf shape in rice have been divided into eight categories related to changes in cell structure, with differences in the number, size and pattern of bulliform cells, mesophyll cells, sclerenchymatous cells, and vascular bundles (Zou et al., 2014). In our study, we isolated and identified a narrow and rolled leaf mutant, *nrl3*. It displays defects in sclerenchymatous cells and a reduced number of vascular bundles.

Sclerenchymatous cells play a crucial role in maintaining normal leaf morphology, and defects lead to loss of support for leaves, resulted in leaf rolling. *SLL1*/*RL9* regulates abaxial sclerenchymatous cell formation to modulate leaf rolling, down-regulate expression of which showed defect in abaxial-side sclerenchymatous cells (Zhang et al., 2009). The *semi-rolled leaf2* (*srl2*) and *narrow and rolled leaf* (*nrl2*) mutant, which is allelic to *nrl3*, showed defective development of the sclerenchymatous cells and rolled leaves (Liu et al., 2016; Zhao et al., 2016). In our study, the *nrl3* mutant showed inward rolling leaves, and statistical analysis showed that the LRI increased about 33% compared to wild-type (Figure 1E). Histological analysis indicated that the abaxial-side sclerenchymatous cells were defective (Figure 2C,F). The *SLL1*/*RL9* was significantly down-regulated in *nrl3* (Figure 5F). Sclerenchymatous cells originate from the differentiation of mesophyll cells via PCD, and are lignified dead cells with thickened secondary cell walls (Zhang et al., 2009). In the process of differentiation, abnormalities in PCD of mesophyll cells can lead to sclerenchymatous cell dysgenesis (Zhang et al., 2009). We found that the *nrl3* mutant had defective sclerenchymatous cells with TE-PCD-related genes *TED2*, *CP*, *C4H*, and *PAL* significantly down-regulated (Figure 5G). This indicates *nrl3* undergoes abnormal PCD during the process of mesophyll cell differentiation to sclerenchymatous cells, which would finally cause the observed defects in the development of sclerenchymatous cells, and produce rolled leaves.

Leaf size is controlled by cell division and expansion, and the obvious feature of narrow leaf mutants in rice is reduction in the number of vascular bundles (Cho et al., 2014). The *narrow leaf* (*nal9*) mutant showed narrow leaves and reduction in the number of large and small vascular bundles, caused by defective cell proliferation (Li et al., 2013). Mutation of *ABNORMAL VASCULAR BUNDLES* (*AVB*), allelic to *nrl3*, gave rise to narrow leaves by affecting the cell division pattern during lateral primordia development (Ma et al., 2017). The (*NARROW LEAF 1*) *NAL1* encodes trypsin-like serine/cysteine protease, down-regulate expression of which in wild-type affects polar auxin transport and narrow leaf (Qi et al., 2008; Jiang et al., 2015). Auxin acts as a signal for cell division, cell expansion and cell differentiation, which play a crucial part in regulating leaf primordia and cell growth in development of leaf (Fujino et al., 2008). In our study, *nrl3* showed narrow leaves and the number of vascular bundles was reduced to only half that of the wild-type, and the transcription expression of *NAL1* was signification alter in *nrl3* (Figure 5F). Thus, the *NRL3* gene may regulate leaf narrowing and the number of vascular bundles by influencing the regulation of auxin, and finally have effection on cell division. Moreover, *OsZHD1*, *OsZHD2*, *Roc5*, and *REL2* that involved in leaf development, the mutants of which caused the changes of the number and the abnormal arrangement of bulliform cells in leaf were resulted in rolling leaves. *SRL1* regulates leaf morphology by negatively regulating the expression of genes encoding vacuolar H^+^-ATPase subunit and H^+^-pyrophosphatase. The transcription expression of these genes showed signification change in *nrl3* (Figure 5F). These results suggested that *NRL3* may synergistically with these genes to regulate leaf morphological development.

Chlorophyll plays an important role in the photosynthesis of plants. In general, chlorophyll content is closely related to photosynthesis competence. However, the “non-functional stay green” mutants, caused by defective degradation of chlorophyll, have much higher chlorophyll content but also lower photosynthetic capacity (Thomas and Howarth, 2000). *Stay Green Rice* (*SGR*) encodes senescence-inducible chloroplast stay-green protein, with a defect in chlorophyll degradation and the mutant showed darker green leaves with loss of photosynthetic competence (Jiang et al., 2007). Both *NON-YELLOW COLORING1* (*NYC1*) and *NYC1-LIKE* (*NOL*) genes encode mutated chlorophyll b reductase, with defective chlorophyll degradation pathways causing leaves to stay green but with decreased photosynthetic competence (Kusaba et al., 2007; Sato et al., 2009). *Non-yellow coloring 3* (*nyc3*) plants exhibited a similar phenotype to the above mutants, also with defective chlorophyll degradation (Morita et al., 2009). In our study, the *nrl3* mutant showed darker green leaves at the tiller stage, with much higher chlorophyll content than wild-type, but the photosynthetic rate was significantly decreased (Figure 1B, 5A and Supplementary Figure S3A). Expression analysis showed the photosynthesis-related genes *NADH*, *RbcS*, *PsaA*, *Cab1R*, and *Cab2R* and chlorophyll degradation related genes *NYC3*, *NYC1*, *NOL*, *PAO*, *SGR*, and *CLH* were all significantly down-regulated in *nrl3*, but the chlorophyll biosynthesis genes were significantly up-regulated, such as *HEME*, *URO*, *CHLH*, *CHLI*, and *PORA* (Supplementary Figure S3C and Figure 5D,E). We hypothesize that the chlorophyll degradation pathway defect leads to the increasing accumulation of non-functional chlorophyll and lower photosynthesis competence, but feedback within the plant accelerates chlorophyll synthesis to compensate for the reduction in functional chlorophyll.

Interestingly, we found that NRL3 protein interacts with VYL/NAL9 protein, an ATP-dependent Clp protease proteolytic subunit affecting leaf size and chloroplast biosynthesis. In higher plants, Clp acts as a protein degradation apparatus to regulating the quantity and quality of protein, which consists of ClpP protein, ClpR protein, ClpT protein, and ClpC protein (Adam et al., 2006; Sakamoto, 2006). Clp protease is a highly conserved protein, containing *ClpP1*, *ClpP4* and other components, regulating plant development and chloroplast biogenesis (Schirmer et al., 1996). Down-regulation of *ClpP1* gave rise to slender leaf shape and chloroplast dysplasia (Shikanai et al., 2001), while knock-down *ClpP4* showed pale-green leaves and abnormal development of mesophyll cell (Zheng et al., 2006). In our study, NRL3 can interacted with VYL/NAL9 directly, but not with ClpP3, ClpP4, ClpP5, and ClpPT, which the five components can form a complex. Previous reports showed that the complex containing VYL, ClpP3, ClpP4, ClpP5, and ClpPT can be functioned as a protein degradation apparatus to regulate chloroplast biogenesis and leaf development (Dong et al., 2013; Li et al., 2013). Based on these data, we speculated that NRL3 may be a component of the complex, and regulating the leaf development and chlorophyll degradation by participate the process of protein degradation, and finally, we propose a complex model by which NRL3 affects leaf development and chlorophyll degradation (Figure 7B), but the accurate regulatory mechanism of this complex needs further investigation.

## Author Contributions

All authors contributed to the creation of the new mutant materials, overall design, and planning of the research.

## Conflict of Interest Statement

The authors declare that the research was conducted in the absence of any commercial or financial relationships that could be construed as a potential conflict of interest.

## Footnotes

1 http://www.clontech.com

2 http://www.v-solid.com/

3 http://rice.plantbiology.msu.edu/cgi-bin/gbrowse/rice/
