# Supplementary material for: Rice Morphogenesis and Chlorophyll Accumulation Is Regulated by the Protein Encoded by NRL3 and Its Interaction With NAL9
Source: Front Plant Sci. 2019 Feb 19;10:175. doi: 10.3389/fpls.2019.00175 (PMC6390494; doi:10.3389/fpls.2019.00175)
Supplement: Supplementary file 1 [file Data_Sheet_1.pdf]

Supplementary Data

Table S1: Primers used in this study.

Primer	Forward:5' to 3'	Reverse:5' to 3'
RM7197	AACGTGGGAATTTCTAGCCC	GTTTTGGGCCTAAACGAGTG
RM14898	ATGTTCAACCTTGTCCCGACTAACC	ATGTTCAACCTTGTCCCGACTAACC
RM3434	AGAGAAATGCCAGCTTTGACTGC	CCAGCTAGGATGTTGAAGGATCG
RM14870	CTCCATGGTGAGGTAGCAAGTGG	CTACCTCATGCTGGCCTTCTAGC
RM2322	GCCGTTAGATCATGATGGGT	GGCTAGTCGACTTGGGTTC
RM14844	AGACATGGCACAAAGAATAGGG	GATTCGTGCAAGCTACATATAGGG
Idel1	TCCAAGGAGTATGACGAAT	ATCTGAATCTCACAACCGA
Idel2	GCGGTAGTAGCCAGAAGCAGAT	AGTACGTGTTGGCGGTGGTG
Idel3	GCGGTAGTAGCCAGAAGCA	ACGTGTTGGCGGTGGTG
NRL3-1390ubi	TTACTTCTGCACTAGGTACCATGGGTTTCATGTCAGCGA	TGGCTAGCGTTAACACTAGTCTGGGCACGATATGCAGCC
PAN580-NRL3	CGGAGCTAGCTCTAGAATGGGTTTCATGTCAGCGA	TGCTCACCATGGATCCCTGGGCACGATATGCAGCC
YN-NRL3	GAGGAGGATCTTCCCGGGATGGGTTTCATGTCAGCGA	ATGCCTGCAGGTCGACCTGGGCACGATATGCAGCC
YC-VYL/NAL9	AGGAGCTCGGTACCCGGGATGGCGCCTATGGCCATCT	ATGGGTACATACTAGTGTATCTTGTTTCCAGCAGG
Crispr/cas9-NRL3	CAGCAGCTCTGCGCCCAAGCTCG	AACCGAGCTTGGGCGCAGAGCTG
Actin	TGTATGCCAGTGGTCGTACCA	CCAGCAAGGTCGAGACGAA
NRL3	GTGAATATGCAGCGAAGAA	TTCCAGAAACTTGGCAATC
HEMA	GATGCAATCACTGCTGGAAAGCGT	CCATCTTGCCAGCACCAATCAACA
HEML	AGAACAAAGGGCAGATTGCTGCTG	TGTTTCGTCAAGTCACGGAGAGCA
HEMB	TGGCATTGTCAGGGAAGATGGAGT	CCAAAGCAGCACGTATTGCTCCAA
HEMC	TCATTCCGAGGGCTATTGGCTTCA	ACACTCTAGTTGGCCAATGGTGGA
HEME	AATGGAGGCTTGCTTGAGCGAATG	TTGTTACCAAGGCGTCTCCTTCCA
URO	AGGCTTCCACTGACAGGTGTTGAT	AAAGAACGCCAGGGTCAACATTCC
HEMF	ACTGACTGCACGATGGCAGT	AGAGATCGAGCCATTCCTTTG

	ATGA	GGT
CHLD	TAGCACAGCTGTCAGAGTGG GTTT	TTGCCAGCCACCTCAAGTATC TCA
CHLH	GCACGGGAACTTGGCGTTTC ATTA	ACATGTCCTGGAGCTGCTTCT CAT
CHL1	AGGGATGCTGAACTCAGGGT GAAA	AAGTAGGACTCACGGAACGC CTTT
CHLM	GCTTCATCTCCACGCAGTTCT ACT	GCAATGACGAATCGAAGACG CACA
DVR	TTCTTCGAGAGGGTGATCAG GGAA	GAAACTGGCAATGGCAGCCA AGAA
PORA	TCGTCGGCCTCGTCTGAGTTT ATT	AGGCCTCTCTCACTGAAAGCT GAA
CHLG	CCAGCCACTGATGAAAGCAG CAAT	AGAGCGCTAATACACTCGCG AACA
Cab1R	AGATGGGTTTAGTGCGACGA G	TTTGGGATCGAGGGAGTATTT
Cab2R	TGTTCTCCATGTTCGGCTTCT	GCTACGGTCCCCACTTCACT
PsaA	GCGAGCAAATAAAACACCTT TC	GTACCAGCTTAACGTGGGGA G
PsbA	CCCTCATTAGCAGATTCGTTT T	ATGATTGTATTCCAGGCAGAG C
RbcL	CTTGGCAGCATTCCGAGTAA	ACAACGGGCTCGATGTGATA
RbcS	TCCGCTGAGTTTTGGCTATTT	GGACTTGAGCCCTGGAAGG
NADH	GGGCAAATCGGATATGTAAT	CGAAGAAACCTGCTAGTGGA
ClpT	AGTCCTATGCACCCACCATTG ACT	AGGAGCAGATGATTGGCGGT TACT
ClpP5	ACATCATCGTTGCCCAGCTGC TAT	ATCGTGTCGAATATGGCCATC CCA
ClpP4	GGGGGATCACTCAGCGCAAC AATGG	ATGGCGAATCGCTTGCCCTTT GTGC
ClpP3	ATCAACTGTCTGCTTTGGACT GGC	AATCCCATCTCTGTGACCTTC CCA
VYL	CAAATCAGGCGTGGCTCT	ATGGCAGGCATTAAAGGT
PI	ATCAAGCCGGAGGTCGCCAT C	CAAGGCAGCGTGTAATCTCC
TED2	AAAGCAATCCAAGCAGCCGA AGACG	CCACGCGGCATCAGACACTCC ATTG
PAL	TACACCGACCACCTCACCCAC A	CGAGCCTCTTCGCCTCCTTCA T
C4H	GGCGAGATCAACCACGACAA CG	GCAACCGCAGCGTCTCCTTCA
CP	CCAACAGGTTCGCCGACCTC	CCGCACGAGCCTTGGTCCTTG

	AC	A
REL2	TGATCATCGTGACTTCACAGG C	TCTACCAGACCACGGACTTGC
RL9	ATTCTTGCAACATGGACGCC	CATTAGCCTCTGTGATTGCC
OSZHD1	CGGACCCCGGTATGGTAG	CGAGAACGAATGCTCTCTCAG
OSZHD2	CCGTGCAGCAGTTCTGCGA	CAGGGTGTGCTTGTTGTTGTG
SRL1	CCCAATTCCTTGGTGACAAGT	TTGCATGAAGAAGACGATGCT
LC2	AGCATCAGCTTTGGACGAGG A	CAGTTGGTGGAATAGAGCCA GAAT
OsBAK1	GAGTTGATCTTGGGAATGCTG C	TGGCCTCCAAGTTGGATGAT
Roc5	CGCAAGAGGAAGAAGCGATA C	GCTCCAGTTGCGTCTTCATC
NYC3	TGCTGCATCCTGTCCACACCT TG	GATGCAAATGATGCAGCAGC TGC
NYC1	GCCATGAATGTCATGCAACA CC	ATCCTCGGAACCAATGTCCTT GC
NOL	GGTGCAACGAAGAGAAGTGT GG	ATTCTGGAGTAGGCTTTCAAG CC
PAO	TGGTAACAGCCAACCTGACT GG	ATCCCAGCAGTTGCACAGAAC G
SGR	CGGTGTCGCACACCATCAAC C	GGAGTGGAAGTAGACCCACA C
CLH	TACCCGGTGGTGGTGTTCTTG	AGTTGATCTCGTCGGTGGTAT C

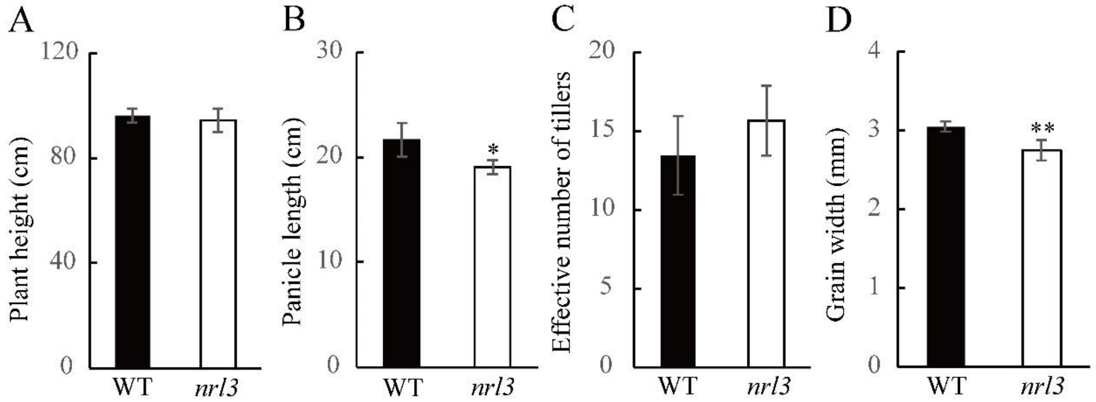

Fig S1. Phenotype of the *nrl3* mutant. (A) to (D) Comparisons of plant height, panicle length, grain width and effective number of tillers between WT and *nrl3*. Data are shown as means ± *SD* (n = 10) in (A-C). Data are means ± *SD* for three biological replicates (D). The asterisk indicates the difference between WT and *nrl3* determined by Student's *t*-test (*P<0.05; **P<0.01)

```
NRL3 : MGFMSAKLFPSCESMCVCCPALRPSSRRPVKRYKKLLAEIFPKTPDGLPNERKIMKLCEYAAKNPLRIPKIAKFLEQRSH : 80
nrl3 : MGFMSAKLFPSCESMCVCCPALRPSSRRPVKRYKKLLAEIFPKTPDGLPNERKIMKLCEYAAKNPLRIPKIAKFLEQRSH : 80
       MGFMSAKLFPSCESMCVCCPALRPSSRRPVKRYKKLLAEIFPKTPDGLPNERKIMKLCEYAAKNPLRIPKIAKFLEQRSH

NRL3 : KELRSAHVNFIKITTEAYSKLLFICKEQMAYFAISLVNVLTELLESKQENIHILGCQTLAKFIYSQVDNTYARNIESLVR : 160
nrl3 : KELRSAHVNFIKITTEAYSKLLFICKEQMAYFAISLVNVLTELLESKQENIHILGCQTLAKFIYSQVDNTYARNIESLVR : 160
       KELRSAHVNFIKITTEAYSKLLFICKEQMAYFAISLVNVLTELLESKQENIHILGCQTLAKFIYSQVDNTYARNIESLVR

NRL3 : KVCVLSRQQGVEHSLLRAASLQCLSAMIWFMKEHSYIFVDFDEIVQSVLENYRVEESAAGDEERHAPQHNWVDEIVRREG : 240
nrl3 : KVCVLSRQQGVEHSLLRAASLQCLSAMIWFMKEHSYIFVDFDEIVQSVLENYRVEESAAGDEERHAPQHNWVDEIVRREG : 240
       KVCVLSRQQGVEHSLLRAASLQCLSAMIWFMKEHSYIFVDFDEIVQSVLENYRVEESAAGDEERHAPQHNWVDEIVRREG

                                           ↓W (TGG, NRL3) → *(TAG, premature termination in nrl3)
NRL3 : RAGLGGGNDVNCNSTAIRLRSARDSSALTREERESPEVWAIIICVQKLAELAKESTTMRRILDPMLSYFDKKKQWAPRQGL : 320
nrl3 : RAGLGGGNDVNCNSTAIRLRSARDSSALTREERESPEV------------------------------------------ : 278
       RAGLGGGNDVNCNSTAIRLRSARDSSALTREERESPEVWAIIICVQKLAELAKESTTMRRILDPMLSYFDKKKQWAPRQGL

NRL3 : ALLVLSDMSYLEKSSGNEQLILTSVIRHLDHKNVLYDPQIKSDMIQTATLLARQLRSRGIAAELVVAGDLCRHLRKTLEA : 400
nrl3 : -------------------------------------------------------------------------------- : -
       ALLVLSDMSYLEKSSGNEQLILTSVIRHLDHKNVLYDPQIKSDMIQTATLLARQLRSRGIAAELVVAGDLCRHLRKTLEA

NRL3 : MESASIEELNLNESLQNFLQDCLLEVVTGINDVRPLYDMMAITLENLPSMPVVARASIGSLLILSHIISLTSMSLNAPMQ : 480
nrl3 : -------------------------------------------------------------------------------- : -
       MESASIEELNLNESLQNFLQDCLLEVVTGINDVRPLYDMMAITLENLPSMPVVARASIGSLLILSHIISLTSMSLNAPMQ

NRL3 : LFPEALLQQILKSMVHPDVDTRVGAHHMFSAVIVQGPSRQRSESDFLYETKKWQSRTTSVFASATALLEKLRREKESLGS : 560
nrl3 : -------------------------------------------------------------------------------- : -
       LFPEALLQQILKSMVHPDVDTRVGAHHMFSAVIVQGPSRQRSESDFLYETKKWQSRTTSVFASATALLEKLRREKESLGS

NRL3 : DKTGNMDDEKEKSISEEENKHVWARKNSAYFSKLVFSFTDRYAALTSSAEEANIVMLTEDQKNQLLSAFWVQAIQTDNTP : 640
nrl3 : -------------------------------------------------------------------------------- : -
       DKTGNMDDEKEKSISEEENKHVWARKNSAYFSKLVFSFTDRYAALTSSAEEANIVMLTEDQKNQLLSAFWVQAIQTDNTP

NRL3 : FNYEAIGHSYSLTVISSRLKDSRNSNNIQFFQLPLSLRSVSLTSNGVLSPSCQRSIFTLATSMLAFAGKVCHITELFDVL : 720
nrl3 : -------------------------------------------------------------------------------- : -
       FNYEAIGHSYSLTVISSRLKDSRNSNNIQFFQLPLSLRSVSLTSNGVLSPSCQRSIFTLATSMLAFAGKVCHITELFDVL

NRL3 : RCFTSCNMDPYLRIGEDLQLYVRLQSDLGNYGSDSDQEIARSVLSDCRTKVGINDQRVLDVVACALCNLTEMDKDVLVKE : 800
nrl3 : -------------------------------------------------------------------------------- : -
       RCFTSCNMDPYLRIGEDLQLYVRLQSDLGNYGSDSDQEIARSVLSDCRTKVGINDQRVLDVVACALCNLTEMDKDVLVKE

NRL3 : LTEMFTPEEVPLFGSNSAFDWANFHVQAFSDESLSFDEECSRTSSVDGGLHESPITNTGSSISKTTMPQSVPRVLGVGQL : 880
nrl3 : -------------------------------------------------------------------------------- : -
       LTEMFTPEEVPLFGSNSAFDWANFHVQAFSDESLSFDEECSRTSSVDGGLHESPITNTGSSISKTTMPQSVPRVLGVGQL

NRL3 : LESALHVAGQVAGASVSTSPLPYGTMTSQCEALGSGTRKKLSSWLVNGHDSTPDNPAPSLPSAQHFIIPKVNSCGFESSI : 960
nrl3 : -------------------------------------------------------------------------------- : -
       LESALHVAGQVAGASVSTSPLPYGTMTSQCEALGSGTRKKLSSWLVNGHDSTPDNPAPSLPSAQHFIIPKVNSCGFESSI

NRL3 : RTTLEPCSAVKLPPASPFDNFLKAAYRAQ : 989
nrl3 : ----------------------------- : -
       RTTLEPCSAVKLPPASPFDNFLKAAYRAQ
```

Fig S2. Protein sequence alignment between NRL3 and nrl3.

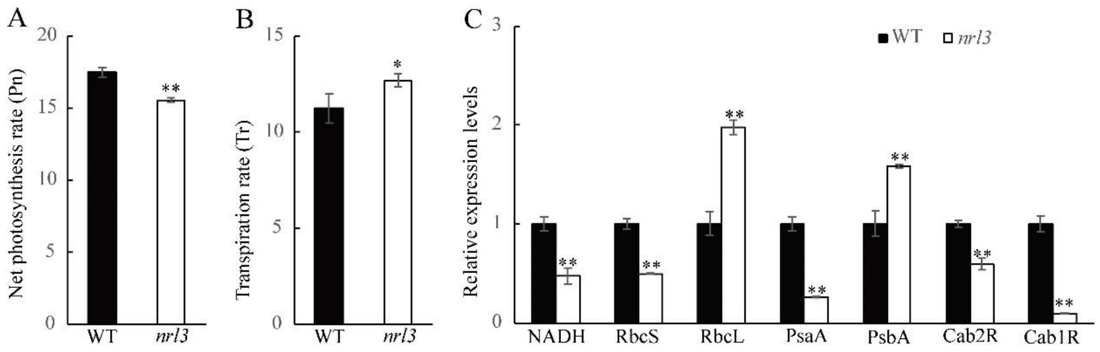

Fig S3. Photosynthetic rate and expression of photosynthesis-related genes in *nrl3*. (A) Net photosynthesis rate in WT and *nrl3*. (B) Transpiration rate in WT and *nrl3*. (C) Expression of photosynthesis-related genes in WT and *nrl3*. Data are means ± *SD* for three biological replicates. The asterisk indicates the difference between WT and *nrl3* determined by Student's *t*-test (**P*<0.05; ***P*<0.01)

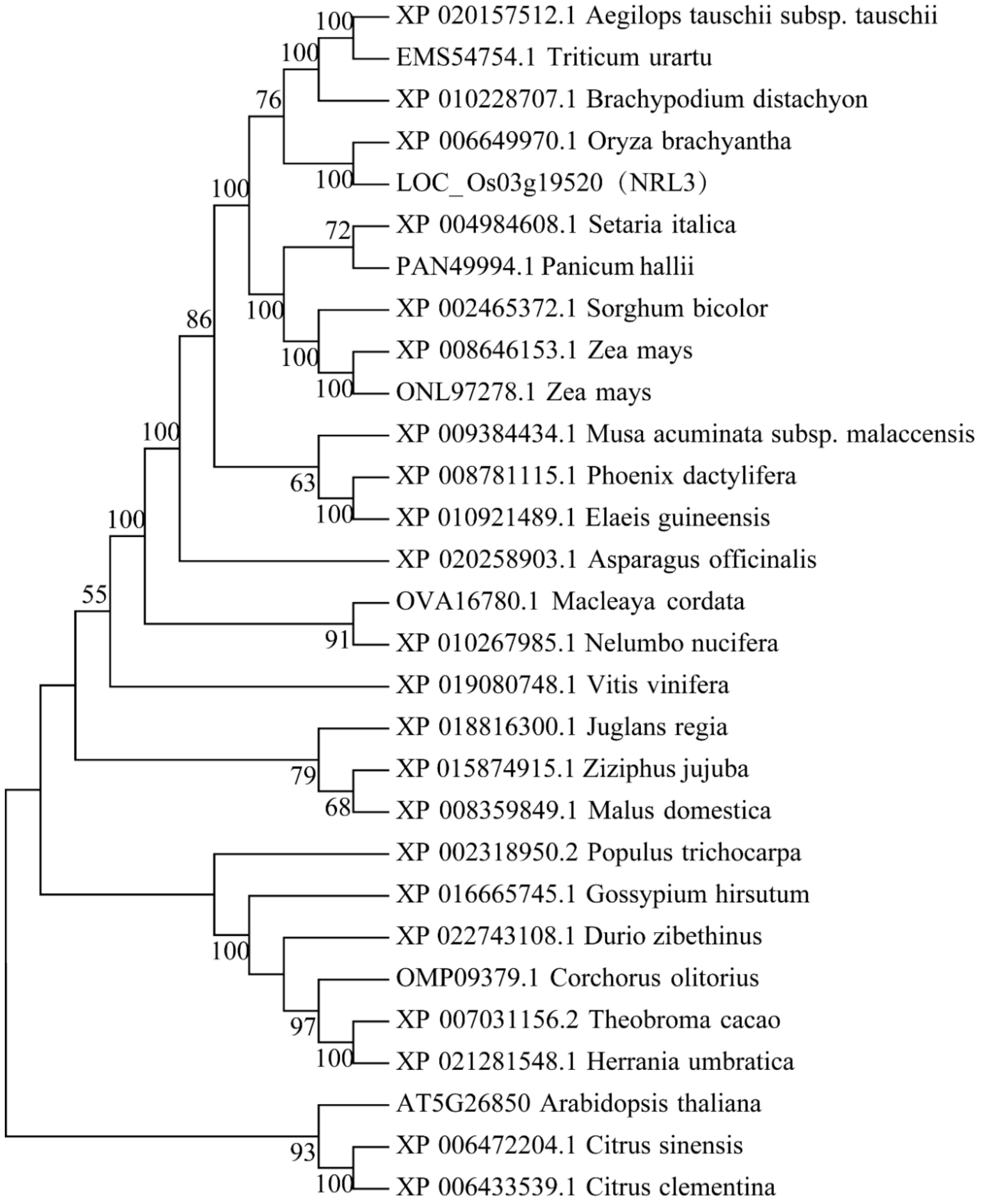

Fig S4. Phylogenetic analysis of NRL3 protein. Evolutionary analyses were conducted in MEGA7 (Kumar *et al*., 2016).

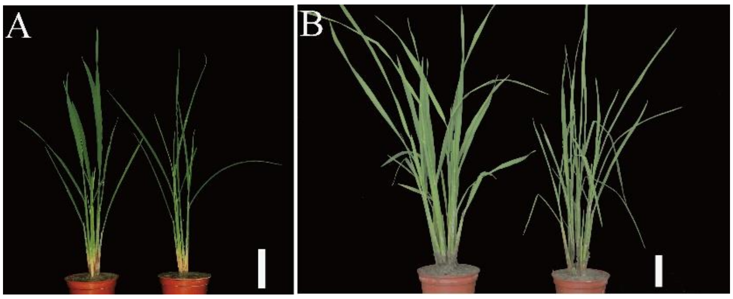

Fig S5. Phenotype of the WT and *nrl3* mutant. (A) The plants of wild-type (WT) (left) and *nrl3*-1 (right) at two-months-old. (B) The plants of wild-type (WT) (left) and *nrl3*-2 (right) at tillering date. Bar: 10 cm (A, B)
